# One Step Synthesis of Tetragonal-CuBi_2_O_4_/Amorphous-BiFeO_3_ Heterojunction with Improved Charge Separation and Enhanced Photocatalytic Properties

**DOI:** 10.3390/nano10081514

**Published:** 2020-08-01

**Authors:** Fang Cai, Ting Zhang, Qiong Liu, Pengran Guo, Yongqian Lei, Yi Wang, Fuxian Wang

**Affiliations:** 1 Guangdong Provincial Key Laboratory of Emergency Test for Dangerous Chemicals, Guangdong Institute of Analysis, Guangdong Academy of Sciences, Guangzhou 510070, China; caifang2687@126.com (F.C.); zhangting@lut.edu.cn (T.Z.); prguo@fenxi.com.cn (P.G.); yqlei@fenxi.com.cn (Y.L.); 2 Guangdong Engineering Technology Research Center of On-line Monitoring of Water Environmental Pollution, Guangdong Institute of Analysis, Guangdong Academy of Sciences, Guangzhou 510070, China; 3 College of Petrochemical Technology, Lanzhou University of Technology, Lanzhou 730050, China; wangyi@lut.cn; 4 State Key Laboratory of Pulp and Paper Engineering, South China University of Technology, Guangzhou 510641, China; liuq6@scut.edu.cn

**Keywords:** tetragonal CuBi_2_O_4_, amorphous BiFeO_3_, visible light absorption, charge carrier recombination, photocatalytic property

## Abstract

Tetragonal CuBi_2_O_4_/amorphous BiFeO_3_ (T-CBO/A-BFO) composites are prepared via a one-step solvothermal method at mild conditions. The T-CBO/A-BFO composites show expanded visible light absorption, suppressed charge recombination, and consequently improved photocatalytic activity than T-CBO or A-BFO alone. The T-CBO/A-BFO with an optimal T-CBO to A-BFO ratio of 1:1 demonstrates the lowest photoluminescence signal and highest photocatalytic activity. It shows a removal rate of 78.3% for the photodegradation of methylene orange under visible light irradiation for 1 h. XPS test after the cycle test revealed the reduction of Bi^3+^ during the photocatalytic reaction. Moreover, the as prepared T-CBO/A-BFO show fundamentally higher photocatalytic activity than their calcinated counterparts. The one-step synthesis is completed within 30 min and does not require post annealing process, which may be easily applied for the fast and cost-effective preparation of photoactive metal oxide heterojunctions.

## 1. Introduction

Bismuth-based multinary metal oxides photocatalysts have attracted wide interest because of their high visible light photocatalytic activity [1]. The valence band of bismuth-based oxides is composed of hybrid orbitals of Bi 6s and O 2p, the presence of Bi 6s slightly above O 2p endows bismuth-based oxides with reduced bandgap for visible light absorption compared to other metal oxide semiconductors [2]. Among the various bismuth-based oxides, multinary metal oxides (Bi_x_M_y_O_z_, M represents other metal) have recently attracted increasing attention since they have shown fewer material limitations, higher controllability and enhanced photoactivity than their binary counterparts [3]. Specifically, CuBi_2_O_4_ has emerged as a promising photocatalytic material because of its suitable bandgap (1.5–1.8 eV) and high photovoltage [4,5,6]. Tetragonal CuBi_2_O_4_ possesses strong visible light absorption and exhibits great potential for photocatalytic degradation of organic pollutants [7,8,9]. Perovskite BiFeO_3_, with special multiferroic property for recycling, is another emerging bismuth-based multinary metal oxide showing great potential for photodegradation of organic pollutants due to its high visible light response [10,11,12]. However, both CuBi_2_O_4_ and BiFeO_3_ suffer from severe carrier recombination due to their intrinsic low polaron conductivity [13,14], which significantly limits their photocatalytic activities [15,16,17,18]. One way to suppress the carrier recombination is to form heterojunctions by combining two semiconductors. Lots of efforts have been paid to combine CuBi_2_O_4_ with TiO_2_ [19], NaTaO_3_ [20], SnO_2_ [21], CeO_2_ [22], g-C_3_N_4_ [5], Fe_2_O_3_ [7], Bi_2_WO_6_ [23], Bi_2_MoO_6_ [24], and incorporate BiFeO_3_ with Bi_2_WO_6_ [25], CuS [26], Ag_3_PO_4_ [27], BiOCl [11], CuO [28], ZnO [12], BiVO_4_ [29], C_3_N_4_ [30]. Such semiconductor combining has been proved to be an effective method to improve the charge separation and photoactivity of CuBi_2_O_4_ and BiFeO_3_. Nevertheless, in most cases the fabrication of the metal oxide-based heterojunction involves at least two steps: the preparation of each semiconductor photocatalyst, followed by the physical mixing and high temperature calcination [31]. The heterojunction is formed during high temperature calcination, which is largely limited by the thermal diffusion property of the semiconductor materials [32].

In this work, the T-CBO/A-BFO heterojunction was successfully formed within 30 min using a one-step solvothermal treatment at 120 °C without the need of post annealing. We demonstrated that the metal oxide photocatalysts do not necessarily need to be completely crystallized for high photocatalytic property. The formation of the T-CuBi_2_O_4_/A-BiFeO_3_ heterojunction resulted in significant improvement in visible light absorption up to 800 nm, enhanced charge separation, and consequently increased photocatalytic properties. We revealed that the oxidation and reduction of Bi^3+^ in the composite during the photocatalytic reaction highlights the importance of protection for the T-CBO/A-BFO. This work provides a facile and cost-effective method for the rapid fabrication of metal-oxides-based photoactive compounds.

## 2. Materials and Methods

### 2.1. Preparation

T-CBO/A-BFO and A-BFO were synthesized by a short-time solvothermal method we have developed previously for the preparation of T-CBO. Specifically the precursor solution of A-BFO was obtained by mixing 15 mL solution of 40 mmol/L Fe(NO_3_)_3_·9H_2_O (99.99%, Aladdin (China) Chemical Co. Ltd., Shanghai, China) in ethanol with 4.5 mL solution of 400 mmol/L Bi(NO_3_)_3_ (99.999%, Aladdin (China) Chemical Co. Ltd., Shanghai, China) in acetic acid. Total of 6.5 mol/L NaOH (98%, Alfa Aesar (China) Chemical Co. Ltd., Shanghai, China) solution was added to the precursor solution under magnetic stirring until pH 14 was reached; the mixture was then solvothermal treated at 120 °C in a 100 mL Teflon-lined steel autoclave for 30 min. For T-CBO/A-BFO composites, the precursor solutions were prepared by mixing Bi(NO_3_)_3_ in acetic acid (400 mmol/L), Cu(NO_3_)_2_·3H_2_O (99.99%, Aladdin (China) Chemical Co. Ltd., Shanghai, China) in ethanol (40 mmol/L), and Fe(NO_3_)_3_·9H_2_O in ethanol (40 mmol/L). Various T-CBO to A-BFO ratios (T-CBO/A-BFO (1:4), T-CBO/A-BFO (1:2), T-CBO/A-BFO (1:1), T-CBO/A-BFO (2:1), T-CBO/A-BFO (3:1), T-CBO/A-BFO (4:1)) could be obtained by adjusting the molar ratios of Cu(NO_3_)_2_·3H_2_O : Fe(NO_3_)_3_·9H_2_O : Bi(NO_3_)_3_ = (1:4:6, 1:2:4, 1:1:3, 2:1:5, 3:1:7, 4:1:9), the details for the preparation of solvothermal solutions with various ion ratios can be found in Table S1. To investigate the effect of post-annealing, the as prepared samples were calcinated in a Muffle furnace at 450 °C for 2 h.

### 2.2. Characterization

A Bruker D8 Advance X-ray diffractometer with Cu Kα radiation was used to analyze the crystal structure of the samples. Raman analysis was conducted using a BWS 465–785 H (BWTEK, Newark, DE, USA) spectrometer with a 785 nm laser source. Scanning electron microscopy (SEM, HITACHI, Tokyo, Japan) and a FEI Titan G2 60-300 transmission electron microscope (TEM, FEI Company, Hillsboro, OR, USA) were applied to characterize the morphologies of the photocatalysts. The chemical states of the elements were tested via an ESCALAB 250XI X-ray photoelectron spectroscopy (XPS, Thermo Fisher Scientific, Waltham, MA, USA). The surface area of the samples was estimated by the TriStar II 3 flex BET model (micrometrics, Norcross, GA, USA). The light absorption properties of the samples were collected on a UV-4100 spectrophotometer (HITACHI, Tokyo, Japan). Photoluminescence (PL) measurements were carried out using a FLS1000 Spectrophotometer (Edinburgh Instruments Ltd., Edinburgh, UK) with an excitation wavelength of 325 nm, fluorescence scanning range is 340 nm to 800 nm, scanning speed is 240 nm/min.

### 2.3. Photocatalytic Activity Measurement

Total of 20 mg photocatalyst was added to 50 mL methyl blue (MB) or methyl orange (MO) solution, and then magnetically stirred in the dark for 30 min to ensure adsorption/desorption equilibrium was achieved. Then, a trace amount of 50 μL H_2_O_2_ (30%) was added to the solution, and the photocatalytic degradation experiment was carried out at 26 °C under the illumination of a LED lamp (λ = 400~900 nm, 100 mW/cm^2^). Total of 2 mL aliquots were extracted from the solution and centrifuged at certain time intervals (5 to 20 min), and the absorption of the aliquots at 654 nm (maximum absorption wavelength of methylene blue) and 464 nm (maximum absorption wavelength of methylene orange) was measured on a UV-vis spectrophotometer. The standard curves were obtained by measuring standard solutions with different dye concentrations, from which the correlation between the absorption and dye concentration was determined. For the stability test, the MO solution was adjusted to the initial concentration and the separated photocatalyst was washed and reused after each cycle of the photocatalytic experiment. The degradation rate was calculated by Equation (1):

Degradation rate (%) = 1−C_t_/C_0_ (1)

where C_0_ is the concentration of the initial dye solution, C_t_ is the remaining concentration of dye solution at a reaction time of t.

## 3. Results and Discussions

### 3.1. Crystal Structure, Morphology, and Composition

**Figure 1:**
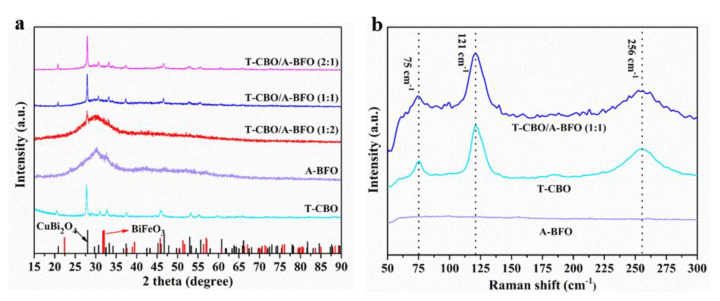
(**a**) XRD patterns of the T-CBO, A-BFO, and T-CBO/A-BFO composites with different composite ratios; (**b**) XRD patterns of the CuBi_2_O_4_, BiFeO_3_, and CuBi_2_O_4_/BiFeO_3_ composite photocatalyst with different composite ratios and after calcination.

The XRD patterns of the as prepared CBO, BFO, and CBO/BFO composites were shown in Figure 1a. For CBO, diffraction peaks at 20.6°, 27.7°, 29.3°, 30.8°, 32.9°, 34.2°, 37.3°, 46.1°, 53.0°, 55.1°, 59.7°, and 65.8° were observed, which can be assigned to the (200), (211), (220), (002), (310), (112), (202), (411), (213), (332), (521), and (413) lattice planes of tetragonal CuBi_2_O_4_ (JCPDF 42-0334), respectively [9,20,22,33,34,35]. For the BFO, only one broad peak appeared at 32.07°, which might be attributed to the (110) peak of perovskite BiFeO_3_ [36,37]. This suggested that the as prepared BiFeO_3_ was mainly amorphous. The CBO/BFO with a CBO to BFO ratio of 1:2 showed a sharp peak at 27.7° in addition to a broad peak at 32.07°, which can be attributed to the (211) peak of tetragonal CuBi_2_O_4_ and the (110) peak of perovskite BiFeO_3_. As the CBO to BFO ratio increased to 1:1 and 2:1, all the peaks of tetragonal CBO appeared, while no characteristic peak of BFO was presented. This suggested that CBO/BFO composites showed the main structure of CBO, and the BFO remained amorphous, this is why they were denoted as T-CBO/A-BFO. To investigate the effect of annealing on the crystal structure, the as-prepared samples were calcined in a muffle furnace at 450 °C for 2 h and the XRD patterns of the samples before and after annealing were displayed in Figure S1a,1b, respectively. The T-CBO maintained the tetragonal structure after the calcination. For the A-BFO, the diffraction peaks at 22.42°, 31.75°, 32.07°, 39.48°, 45.75°, 51.31°, 51.74°, 56.97°, and 67.07° appeared after calcination, which can be assigned to the (012), (104), (110), (202), (024), (116), (122), (214), and (220) lattice planes of BiFeO_3_ (JCPDF 86-1518), respectively [36,37]. In addition, the diffraction peaks at 28.2°, 31.6°, 33.1°, 44.1°, 46.8°, 49.1°, and 53.5° were obviously observed, which can be attributed to the (400), (420), (332), (532), (541), (444), and (721) lattice planes of Bi_25_FeO_40_ (JCPDF 86-0368), respectively [36]. It has been reported that pure phase BiFeO_3_ was difficult to prepare via hydrothermal methods, and usually multiphase was obtained (e.g., Bi_25_FeO_40_, Bi_2_Fe_4_O_9_) [36]. However, after high temperature calcination, the T-CBO/A-BFO composites showed CBO and BFO diffraction peaks without the appearance of Bi_25_FeO_40_ or Bi_2_Fe_4_O_9_. This implied that rapid grown CBO could provide nucleation center for BFO. Raman spectroscopy was carried out to investigate the vibration modes of the samples, and the results are shown in Figure 1b. No obvious peak was found in the Raman spectra of A-BFO, which further confirmed that the as prepared A-BFO was amorphous as demonstrated in the XRD results. For T-CBO, three main Raman bands of tetragonal CuBi_2_O_4_ at 76, 121, and 256 cm^−1^ were observed. The band at 76 cm^−1^ corresponds to the B_2g_ mode of the in-plane bending vibration of the Bi rhombohedra. The strong peak at 123 cm^−1^ can be assigned to the *A*_1g_ mode of the translational vibration of the CuO_4_ plane along the Z-axis. The broad peak at 256 cm^−1^ is attribute to the rotation of two stacked CuO_4_ squares in opposite directions [38,39,40]. The T-CBO/A-BFO (1:1) showed main peaks of CuBi_2_O_4_ at the same position as T-CBO, indicating that the T-CBO/A-BFO (1:1) maintained the main structure of T-CBO.

**Figure 2:**
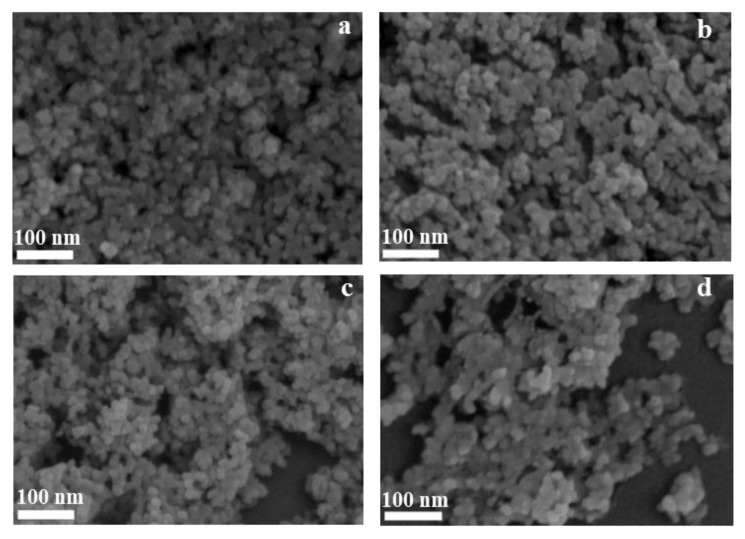
SEM images of (**a**) A-BFO; (**b**) T-CBO/A-BFO (1:2); (**c**) T-CBO/A-BFO (1:1); (**d**) T-CBO/A-BFO (2:1).

SEM was performed to investigate the morphologies of T-CBO, A-BFO, and T-CBO/A-BFO composites, the images are shown in Figure 2. It can be observed that pure T-CBO photocatalysts (Figure S2) were formed by the self-assembling of nanorod arrays with a dumbbell-like morphology, more details of the T-CBO can be found in our previous report [9]. The A-BFO photocatalyst (Figure 2a) showed an irregular granular morphology with small particle size. Figure 2a–d showed the morphologies of T-CBO/A-BFO composites with different composite ratios. The T-CBO/A-BFO composites showed similar irregular granular morphology to A-BFO, and the particle size was obviously smaller than that of pure T-CBO.

**Figure 3:**
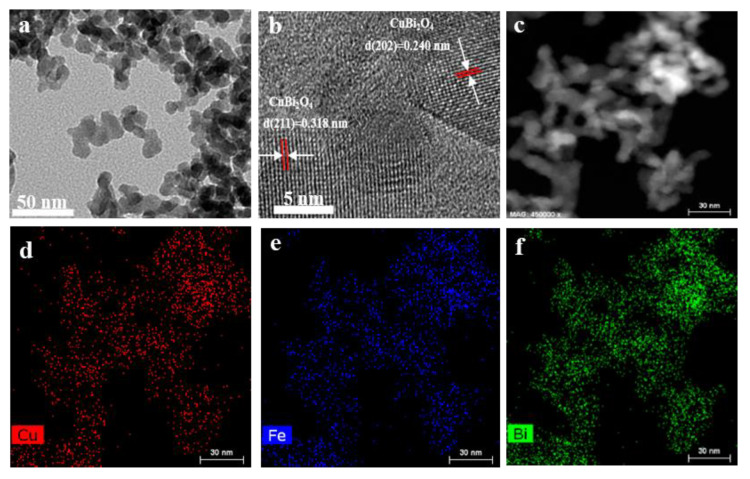
(**a**) TEM image; (**b**) HRTEM image; (**c**) TEM image of the selected mapping area and the corresponding elemental mapping of (**d**) Cu, (**e**) Fe, (**f**) Bi of the T-CBO/A-BFO (1:1). The arrows pointed to the interplanar spacing for different CBO planes.

The fine structure of T-CBO/A-BFO (1:1) composite was characterized by HRTEM. The nanoparticles of T-CBO/A-BFO (1:1) composites were observed in Figure 3a. The well-ordered lattice fringes were presented in Figure 3b, in which an interplanar spacing of 0.318 nm and 0.240 nm were observed, corresponding to the (211) and (202) plane of T-CBO, respectively [7,41,42]. No obvious A-BFO lattice fringes was found, which is consistent with the XRD and Raman results. This further confirmed the amorphous nature of BiFeO_3_ in the T-CBO/A-BFO (1:1) composite. The microstructure and elemental distribution of the T-CBO/A-BFO (1:1) composites photocatalyst were further investigated by HAADF-STEM and energy dispersive-ray spectroscopy (EDS). In the mapping area (Figure 3c), the Cu, Fe, and Bi were uniformly distributed in the T-CBO/A-BFO (1:1) composite, as shown in Figure 3d–f.

**Figure 4:**
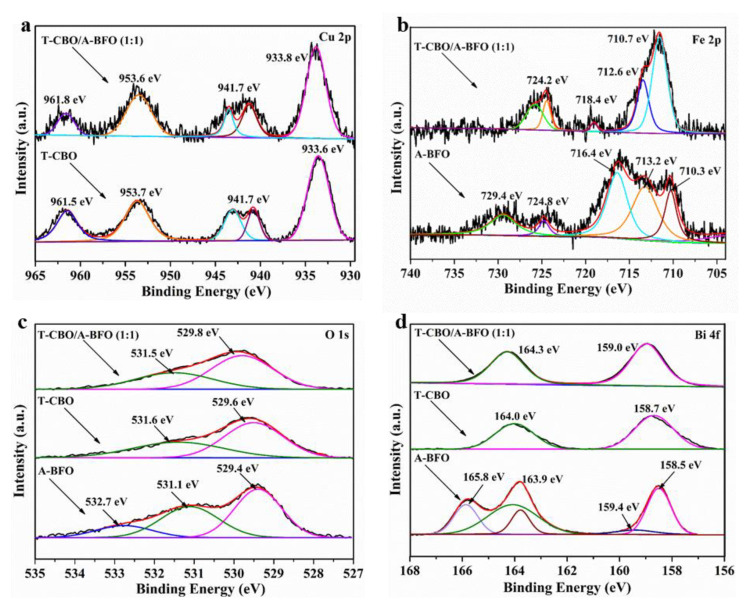
XPS high-resolution spectra of (**a**) Cu 2p, (**b**) Fe 2p, (**c**) O 1s, and (**d**) Bi 4f for the T-CBO, A-BFO, and T-CBO/A-BFO (1:1). The arrow pointed to the curves of different samples, and specific XPS peaks in case of overlapping.

The chemical composition and elemental valence of T-CBO, A-BFO, and T-CBO/A-BFO (1:1) composite were analyzed by XPS, and the results are shown in Figure 4. The XPS survey spectra for the Cu, Bi, Fe, and O are displayed in Figure S3. All the XPS spectra were calibrated by the binding energy of C 1s at 284.8 eV. As shown in Figure 4a, T-CBO and T-CBO/A-BFO (1:1) showed two main peaks at around 933.8 eV and 953.6 eV and shake-up satellites at approximately 941.7 eV and 961.8 eV, which could be assigned to Cu 2p_3/2_ and Cu 2p_1/2_ binding energies of Cu^2+^, respectively [38,42,43,44]. In the A-BFO, two peaks at 710.3 eV and 724.8 eV were observed (Figure 4b), corresponding to the Fe 2p_3/2_ and Fe 2p_1/2_ peaks of Fe^3+^. In addition, two satellite peaks at 716.4 eV and 729.4 eV appeared in the A-BFO, which can be assigned to the Fe 2p_3/2_ and Fe 2p_1/2_ satellite of Fe^2+^, respectively. This suggests the co-existence of Fe^3+^ and Fe^2+^ in the A-BFO. Whereas in the T-CBO/A-BFO (1:1) composite, the peaks at 710.7 eV and 724.2 eV correspond to Fe 2p_3/2_ and Fe 2p_1/2_ of Fe^3+^, respectively, and the small satellite peak at 718.4 eV further confirmed the existence of Fe^3+^ [7,37]. No satellite peaks of Fe^2+^ was found in the T-CBO/A-BFO (1:1) composite, suggesting different valence bond mechanisms of A-BFO when it was co-synthesized with the T-CBO. In Figure 4c, the peak for O-mental bounds appeared at 529.4, 529.6, and 529.8 for the A-BFO, T-CBO, and T-CBO/A-BFO (1:1) respectively. The positive shift in the T-CBO/A-BFO (1:1) was due to the increase in binding energy of O-mental in the heterojunction. The peaks at around 531.5 eV can be assigned to the chemisorbed oxygen [45,46], and the peak at 532.7 eV in the A-BFO suggested the formation of metal-OH groups on the surface [47]. In Figure 4d, the T-CBO, A-BFO, and T-CBO/A-BFO (1:1) all showed peaks at around 158.7 eV and 164.0 eV, which can be assigned to Bi 4f 7/2 and Bi 4f 5/2 of Bi^3+^, respectively. The positive shift in the T-CBO/A-BFO (1:1) was attributed to the increasing bounding energy of Bi^3+^ with the surrounding O atoms due to the formation of the heterojunction. The A-BFO, in addition, had two peaks at 159.4 eV and 165.8 eV, which could be attributed to the Bi 4f 7/2 and Bi 4f 5/2 of Bi^5+^ [48]. This implied the co-existence of Bi^3+^ and Bi^5+^ in the A-BFO.

**Figure 5:**
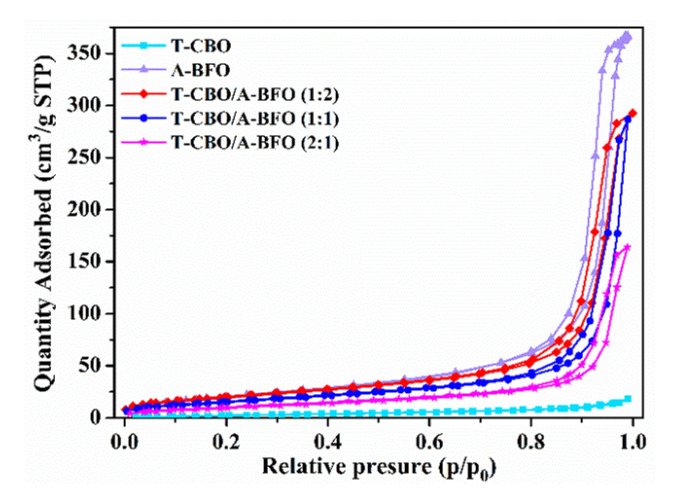
N_2_ adsorption-desorption isotherms of the T-CBO, A-BFO, T-CBO/A-BFO (2:1), T-CBO/A-BFO (1:1), and T-CBO/A-BFO (2:1) composites.

In general, photocatalysts with high specific surface area and large pore size are expected to provide more photocatalytic active sites, which is beneficial for improving the photocatalytic activity. To elucidate the effect of specific area, BET measurements were carried out. The N_2_ adsorption-desorption curves with T-CBO, A-BFO, and T-CBO/A-BFO with different composite ratios were displayed in Figure 5. It can be seen that all the samples had isotherms of type IV, indicating the presence of mesopores (2–50 nm). The isotherms exhibited H3 hysteresis loops at a high relative pressure range from 0.8 to 1.0, suggesting the presence of slit-like pores.

The BET surface area, pore volume, and average pore size of the samples are summarized in Table 1. The instrument for BET test (micrometrics, TriStar II 3 flex, THE UNITED STATES OF AMERICA) could extend specific surface area, pore volume, and average pore size measurements to as low as 0.001 m^2^/g, 4 × 10^−6^ cm^3^/g, and 0.01 nm, respectively. The uncertainties of the BET results were obtained by repeating the measurements three times, from which the standard deviation was calculated. The T-CBO and A-BFO showed a BET surface area of 11.60 m^2^/g and 80.39 m^2^/g, respectively. The surface area of the T-CBO/A-BFO composite was in between T-CBO and A-BFO, and increased from 38.95 m^2^/g to 74.18 m^2^/g as the T-CBO to A-BFO ratio decreased from 2:1 to 1:2. Specifically, the T-CBO/A-BFO (1:1) showed a surface area of 58.86, which is relatively higher compared to the reported values (7.2–28.1 m^2^/g) for BFO and its compounds [49,50].

### 3.2. Optical and Electronic Properties

**Figure 6:**
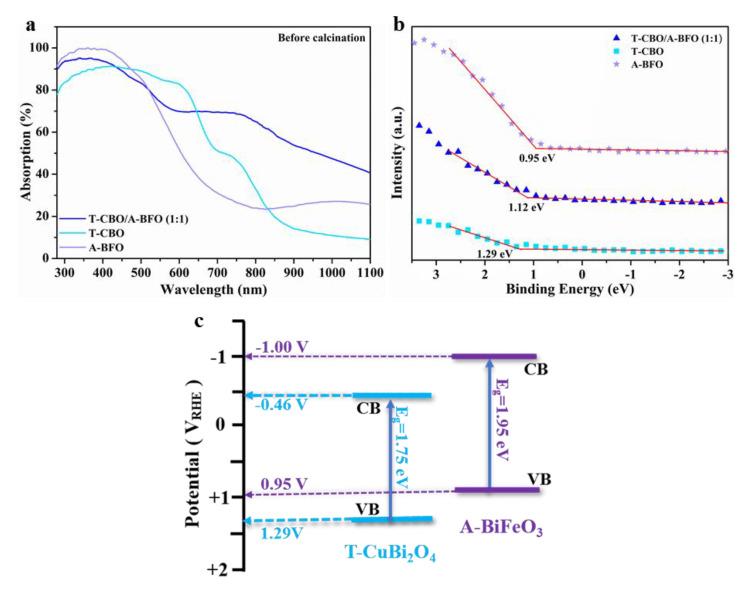
(**a**) UV-vis diffuse reflectance spectra; (**b**) XPS VB spectra of the T-CBO, A-BFO and T-CBO/A-BFO (1:1); (**c**) schematic energy band diagram.

The optical absorption properties of the T-CBO, A-BFO, and T-CBO/A-BFO (1:1) were investigated by the UV-vis diffusive reflectance analyzer, and the spectra are shown in Figure 6a. UV-vis spectra for T-CBO/A-BFO composites with other T-CBO to A-BFO ratios were provided in Figure S4a, and the corresponding Tauc plots could be found in Figure S4b.

The A-BFO could effectively absorb light under 500 nm, while the T-CBO exhibited wide visible light absorption up to 800 nm. The T-CBO/A-BFO composites showed significant higher absorption and extended absorption region compared with the A-BFO. The bandgap of the T-CBO and A-BFO were estimated to be 1.75 eV, 1.95 eV from the Tauc plot (Figure S4b), and their valence band edge was determined to be 1.29 V and 0.95 V from the XPS VB spectra (Figure 6b). Based on the valence band positions and bandgaps of the T-CBO and A-BFO, we have tried to sketch an energy band diagram of the T-CBO/A-BFO composite, as shown in Figure 6c. The band edges of A-BFO are located slightly higher than those of T-CBO, such band alignment is beneficial for forming T-CBO/A-BFO heterojunction with improved charge separation efficiency. In the T-CBO/A-BFO heterojunction, the photo-generated electrons on the CB of A-BFO tend to move onto the CB of T-CBO, while the photo-generated holes on the VB of T-CBO can be effectively transferred to the VB of A-BFO.

**Figure 7:**
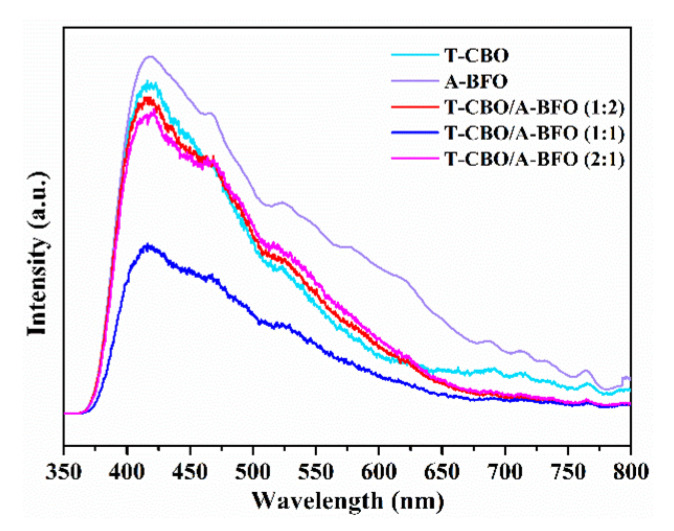
PL spectra of T-CBO, A-BFO, T-CBO/A-BFO (1:2), T-CBO/A-BFO (1:1), T-CBO/A-BFO (2:1).

To further investigate the carrier recombination and charge transfer properties, the T-CBO, A-BFO, and T-CBO/A-BFO (1:2), T-CBO/A-BFO (1:1), T-CBO/A-BFO (2:1) were evaluated by photoluminescence (PL) with an excitation wavelength of 325 nm and the results are shown in Figure 7. All the samples showed emission peak at about 420 nm. Both T-CBO and A-BFO exhibited strong emission peak, while a decrease in emission signal was observed in all the T-CBO/A-BFO composites, indicating inhibited carrier recombination in the composites. This confirmed that the formation of T-CBO/A-BFO heterojunction benefited the charge transfer between the T-CBO and A-BFO, as has been demonstrated in Figure 6c. The PL emission intensity decreased significantly as the T-CBO to A-BFO ratio increased from 1:2 to 1:1, further increasing the ratio to 2:1 resulted in a sharp increase in PL emission. This implied that recombination of photo-generated electron-hole pairs was effectively prohibited in the T-CBO/A-BFO composites and the optimal T-CBO to A-BFO ratio is 1:1.

### 3.3. Photocatalytic Properties

**Figure 8:**
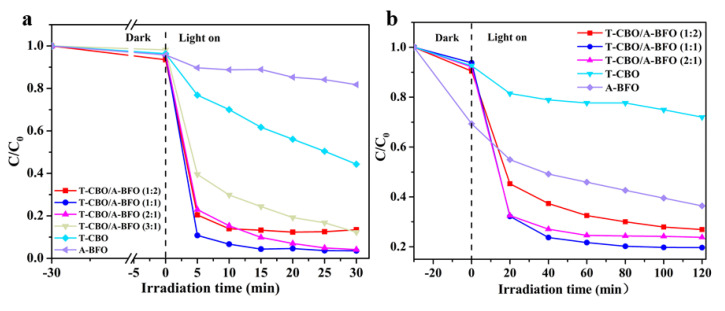
photocatalytic degradation of (**a**) methylene blue; (**b**) methyl orange. C_0_ is the concentration of the initial dye solution, C_t_ is the remaining concentration of dye solution at a reaction time of t, C_t_/C_0_ is the residual ratio of the dye in the solution, the degradation rate equals 1 − C_t_/C_0_.

The photocatalytic performance of the as-prepared samples was evaluated for the degradation of MB and MO under visible light. As shown in Figure 8, the MO concentration obviously decreased under dark, especially for the dye solution with A-BFO added as the photocatalyst. This is probably due to the strong physical absorption of dye on the surface of BiFeO_3_, which has been reported previously [51]. Among all our samples, A-BFO showed the highest BET surface area (80.39 m^2^/g, see Table 1). Previous study demonstrated that larger surface area could lead to higher absorption of dye in the dark [52]. After 30 min of visible light irradiation, the degradation rates of MB by the T-CBO and A-BFO photocatalysts were 56% and 19%, respectively. Obviously, the T-CBO/A-BFO composites showed increased photocatalytic property than the T-CBO and A-BFO alone. Based on the aforementioned results, we tried to elucidate the correlation between the crystal structure, specific surface area, light absorption property, carrier transfer of the samples with their photocatalytic properties. To investigate the effect of crystal structure, the as-prepared samples were calcined in a muffle furnace at 450 °C for 2 h and the photocatalytic property was tested. Tetragonal CBO and perovskite BFO were obtained after calcination (Figure S1b), which were commonly regarded as the photoactive phases. However, in our results the well crystalized tetragonal-CBO/perovskite-BFO composite after calcination showed substantially lower photoactivity than the as-prepared T-CBO/A-BFO (Figure S5), indicating that better crystallinity did not lead to higher photoactivity. With respect to the surface area, in our study the A-BFO processed the highest BET surface area (Table 1), but it exhibited the lowest photoactivity (Figure 8a). Therefore, specific surface area could not be the main determinant for the photocatalytic performance. Herein, the improved photoactivity of the CBO/A-BFO composite was mainly attributed to the extended visible light absorption (Figure 6a) and improved charge transfer of the T-CBO/A-BFO heterojunction (Figure 6). The photoactivity of the CBO/A-BFO composite increased as the T-CBO to A-BFO ratio increases from 1:2 to 1:1. Further increasing the ratio from 1:1 to 2:1 resulted in a decrease in photoactivity, which is probably due to the sharp increase in carrier recombination as demonstrated in Figure 7. The same trend in photoactivity was also found for the degradation of MO, as shown in Figure 8b. Among the T-CBO/A-BFO composites with various T-CBO to A-BFO ratios, the T-CBO/A-BFO (1:1) composite exhibited the best photocatalytic performance and it could photodegrade 97% MB within 30 min and 80% MO within 80 min. An interesting observation about the T-CBO/A-BFO 1:2 and 2:1 was that they showed PL signal almost as high as the T-CBO or A-BFO alone, whereas their photocatalytic activity were close to that of T-CBO/A-BFO 1:1. This suggested that other factors (e.g., light absorption, surface area) played an important role in the photocatalytic activity of the T-CBO/A-BFO 1:2 and 2:1, even though they might not be the main determinants. Specifically, T-CBO/A-BFO 2:1 possessed considerably higher visible light absorption in the range of 520–800 nm compared to T-CBO/A-BFO 1:1, as shown in Figure S4a. T-CBO/A-BFO 1:2 exhibited much higher specific surface area (74.18 m^2^/g) than the T-CBO/A-BFO 1:1 (58.86 m^2^/g).

Moreover, we also prepared T-CBO and A-BFO composite by simply physically mixing T-CBO and A-BFO at a ratio of 1:1, and the photocatalytic performance was tested (Figure S6). The experimental results showed that the degradation rate of MO by the physically mixed T-CBO/A-BFO was slightly higher than that of T-CBO or A-BFO alone, but still much lower than that of the one-step synthesized T-CBO/A-BFO (1:1) composite. This indicated that a solid heterojunction T-CBO/A-BFO (1:1) was formed during the solvothermal reaction, leading to improved charge carrier separation and enhanced photocatalytic performance.

### 3.4. Stability of the T-CBO/A-BFO (1:1) Composite

**Figure 9:**
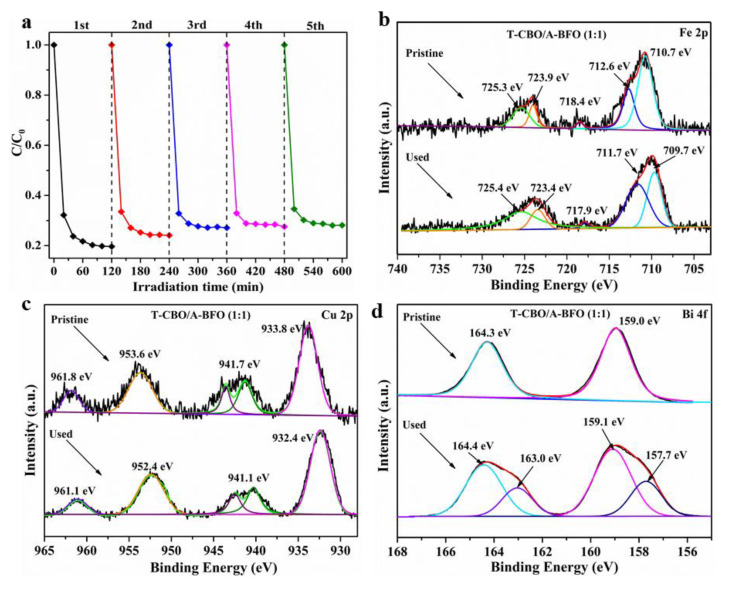
(**a**) Five-cycle test of T-CBO/A-BFO (1:1) composite photocatalyst for the degradation of methyl orange; (**b**) Fe 2p, (**c**) Cu 2p, and (**d**) Bi 4f XPS spectra of the T-CBO/A-BFO (1:1) composite before and after the stability test. The arrows pointed to the curves of pristine and used samples and specific XPS peaks in case of overlapping.

Eventually, the stability of the T-CBO/A-BFO (1:1) composite was evaluated by repeating photodegradation experiments under visible light irradiation for 5 cycles. As illustrated in Figure 9a, after five cycles of experiments, the decomposition rate of MO by the T-CBO/A-BFO (1:1) slightly decreased by 8.4%, demonstrating the relatively high stability of the as-prepared T-CBO/A-BFO (1:1) composite photocatalyst. We note that the decrease may be partially due to the loss of the sample powder during the separation and cleaning processes. XPS of Fe, Cu, and Bi was performed before and after the stability test, as shown in Figure 9b–d. No significant change was found for Fe 2p (Figure 9b) and Cu 2p (Figure 9c) before and after the stability test. However, in the Bi 4f spectra, two new peaks at lower binding energy of 157.7 and 163.0 appeared after the cycle experiments, suggesting the reduction of Bi^3+^ into lower valence states such as Bi^2.75^ [53], Bi^2+^ [54] during the photocatalytic test. Previously we have demonstrated that the T-CBO was stable after 5 cycles photodegradation test [9], therefore the reduction of Bi^3+^ was presumably taking place in the A-BFO of the T-CBO/A-BFO composite. This may be part of the reason for the decreased photocatalytic property in the cycle experiment. Our results highlight the importance of preventing Bi^3+^ from reduction to further improve the stability of the T-CBO/A-BFO composite.

## 4. Conclusions

In this study, a T-CBO/A-BFO composite was prepared using a facile one-step method. A 30-min solvothermal treatment at 120 °C resulted in T-CBO/A-BFO composites with extended visible light absorption region, lower charge recombination and consequently higher photocatalytic performance compared to T-CBO or A-BFO alone. The photoluminance intensity of the CBO/A-BFO composite decreased as the T-CBO to A-BFO ratio increases from 1:2 to 1:1. Further increasing the ratio from 1:1 to 2:1 resulted in a sharp increase in photoluminance intensity. The CBO/A-BFO (1:1) composite showed the lowest photoluminance intensity, indicating suppressed carrier recombination and improved the charge transfer in the heterojunction. The CBO/A-BFO (1:1) composite had a high BET surface area of 58.86 m^2^/g, and exhibited wide visible light absorption up to 800 nm. It could degrade 95.6% MB within 15 min, and 78.3% MO within 1 h under visible light illumination. The reduction of Bi^3+^ was observed after the photocatalytic stability test. The CBO/A-BFO (1:1) composite prepared by the one-step method showed significantly higher photocatalytic activity than the physically mixed T-CBO and A-BFO with a ratio of 1:1, which demonstrates the advantage of the one-step synthesized T-CBO/A-BFO heterojunction. Moreover, the as prepared CBO/A-BFO (1:1) composite exhibited substantially higher photocatalytic performance than its post annealed counterpart. The short-time synthesis method was carried out at mild reaction conditions and high temperature calcination was not required. This work provides an easy and cost-effective route for the fast preparation of multinary metal oxide-based heterojunctions for photocatalytic applications.

## Figures and Tables

**Table 1:** BET surface area, pore volume, and average pore size of the samples.

Samples	T-CBO	T-CBO/A-BFO (2:1)	T-CBO/A-BFO (1:1)	T-CBO/A-BFO (1:2)	A-BFO
BET (m^2^/g)	11.60 ± 0.39	38.95 ± 0.47	58.86 ± 0.48	74.18 ± 0.66	80.39 ± 0.63
Pore volume (cm³/g)	0.02 ± 0.01	0.19 ± 0.03	0.27 ± 0.08	0.41 ± 0.02	0.57 ± 0.01
Average pore size (nm)	6.82 ± 0.94	17.56 ± 2.25	21.78 ± 2.60	17.59 ± 1.19	28.22 ± 1.05

## Supplementary Materials

The following are available online at https://www.mdpi.com/2079-4991/10/8/1514/s1. Figure S1: XRD patterns of the T-CBO, A-BFO, and T-CBO/A-BFO composites (**a**) before annealing; (**b**) after annealing at 450 °C for 2 h. Figure S2: SEM images of the T-CBO. Figure S3: XPS survey spectra of Cu 2p, Fe 2p, O 1s, Bi 4f of the T-CBO, A-BFO and T-CBO/A-BFO (1:1). Figure S4: (**a**) UV-vis diffuse reflectance spectra; (**b**) corresponding Tauc plots of the T-CBO, A-BFO, and T-CBO/A-BFO composites. Figure S5: Photodegradation of MB by post annealed CBO, BFO, and CBO/BFO composites. Figure S6: Photodegradation of MO by T-CBO/A-BFO (1:1) and physical mixed T-CBO and A-BFO at a ratio of 1:1.

## References

[1] Meng X., Zhang Z. (2016). Bismuth-based photocatalytic semiconductors: Introduction, challenges and possible approaches. J. Mol. Catal. A Chem..

[2] He R., Cao S., Zhou P., Yu J. (2014). Recent advances in visible light Bi-based photocatalysts. Chin. J. Catal..

[3] Abdi F.F., Berglund S.P. (2017). Recent developments in complex metal oxide photoelectrodes. J. Phys. D Appl. Phys..

[4] Kang D., Hill J.C., Park Y., Choi K.-S. (2016). Photoelectrochemical properties and photostabilities of high surface area CuBi_2_O_4_ and Ag-doped CuBi_2_O_4_ photocathodes. Chem. Mater..

[5] Guo F., Shi W., Wang H., Huang H., Liu Y., Kang Z. (2017). Fabrication of a CuBi_2_O_4_/g-C_3_N_4_ p–n heterojunction with enhanced visible light photocatalytic efficiency toward tetracycline degradation. Inorg. Chem. Front..

[6] Wang F., Chemseddine A., Abdi F.F., Krol R.v.d., Berglund S.P. (2017). Spray pyrolysis of CuBi_2_O_4_ photocathodes: Improved solution chemistry for highly homogeneous thin films. J. Mater. Chem. A..

[7] Li M.Y., Tang Y.-B., Shi W.-L., Chen F.-Y., Shi Y., Gu H.C. (2018). Design of visible-light-response core–shell Fe_2_O_3_/CuBi_2_O_4_ heterojunctions with enhanced photocatalytic activity towards the degradation of tetracycline: Z-scheme photocatalytic mechanism insight. Inorg. Chem. Front..

[8] Chen X., Dai Y., Guo J. (2015). Hydrothermal synthesis of well-distributed spherical CuBi_2_O_4_ with enhanced photocatalytic activity under visible light irradiation. Mater. Lett..

[9] Wang Y., Cai F., Guo P., Lei Y., Xi Q., Wang F. (2019). Short-Time Hydrothermal Synthesis of CuBi_2_O_4_ Nanocolumn Arrays for Efficient Visible-Light Photocatalysis. Nanomaterials.

[10] Lam S.M., Sin J.C., Mohamed A.R. (2017). A newly emerging visible light-responsive BiFeO_3_ perovskite for photocatalytic applications: A mini review. Mater. Res. Bull..

[11] Shang J., Chen H., Chen T., Wang X., Feng G., Zhu M., Yang Y., Jia X. (2019). Photocatalytic degradation of rhodamine B and phenol over BiFeO_3_/BiOCl nanocomposite. Appl. Phys. A.

[12] Sahni M., Kumar D., Chauhan S., Singh M., Kumar N. (2020). Study of structural, optical and photocatalytic activity of Sm and Ni doped BiFeO_3_ (BFO) and BFO@ZnO nanostructure. Mater. Today Proc..

[13] Sivula K., Roel V.D.K. (2016). Semiconducting materials for photoelectrochemical energy conversion. Nat. Rev. Mater..

[14] Henrich V.E., Cox P.A. (1994). The Surface Science of Metal Oxides.

[15] Wang F., Septina W., Chemseddine A., Abdi F.F., Friedrich D., Bogdanoff P., Krol R.v.d., Tilley S.D., Berglund S.P. (2017). Gradient self-doped CuBi_2_O_4_ with highly improved charge separation efficiency. J. Am. Chem. Soc..

[16] Sheu Y.M., Trugman S.A., Xiong J., Park Y.S., Lee S., Yi H.T., Cheong S.W., Jia Q.X., Taylor A.J., Prasankumar R.P. (2013). Ultrafast carrier dynamics and radiative recombination in multiferroic BiFeO_3_ single crystals and thin films. Proceedings of EPJ Web of Conferences.

[17] Sheikh M.S., Ghosh D., Bhowmik T.K., Dutta A., Bhattacharyya S., Sinha T.P. (2020). When multiferroics become photoelectrochemical catalysts: A case study with BiFeO_3_/La_2_NiMnO_6_. Mater. Chem. Phys..

[18] Berglund S.P., Abdi F.F., Bogdanoff P., Chemseddine A., Friedrich D., Krol R.v.d. (2016). Comprehensive Evaluation of CuBi_2_O_4_ as a photocathode material for photoelectrochemical water splitting. Chem. Mater..

[19] Wei L., Shifu C., Sujuan Z., Wei Z., Huaye Z., Xiaoling Y. (2009). Preparation and characterization of p–n heterojunction photocatalyst p-CuBi_2_O_4_/n-TiO_2_ with high photocatalytic activity under visible and UV light irradiation. J. Nanopart. Res..

[20] Deng Y., Chen Y., Chen B., Ma J. (2013). Preparation, characterization and photocatalytic activity of CuBi_2_O_4_/NaTaO_3_ coupled photocatalysts. J. Alloys Compd..

[21] Abdelkader E., Nadjia L., Ahmed B. (2015). Preparation and characterization of novel CuBi_2_O_4_/SnO_2_ p–n heterojunction with enhanced photocatalytic performance under UVA light irradiation. J. King Saud Univ. Sci..

[22] Elaziouti A., Laouedj N., Bekka A., Vannier R.-N. (2015). Preparation and characterization of p–n heterojunction CuBi_2_O_4_/CeO_2_ and its photocatalytic activities under UVA light irradiation. J. King Saud Univ. Sci..

[23] Yuan X., Shen D., Zhang Q., Zou H., Liu Z., Peng F. (2019). Z-scheme Bi_2_WO_6_/CuBi_2_O_4_ heterojunction mediated by interfacial electric field for efficient visible-light photocatalytic degradation of tetracycline. Chem. Eng. J..

[24] Shi H., Fan J., Zhao Y., Huc X., Zhang X., Tang Z. (2020). Visible light driven CuBi_2_O_4_/Bi_2_MoO_6_ p-n heterojunction with enhanced photocatalytic inactivation of E. coli and mechanism insight. J. Hazard. Mater..

[25] Samran B., Saranyoo C. (2019). Highly enhanced photoactivity of BiFeO_3_/Bi_2_WO_6_ composite films under visible light irradiation. Physica B.

[26] Mishra] B.G. (2018). Photocatalytic degradation of alachlor using type-II CuS/BiFeO_3_ heterojunctions as novel photocatalyst under visible light irradiation. Chem. Eng. J..

[27] Di L., Yang H., Xian T., Chen X. (2018). Facile Synthesis and Enhanced Visible-Light Photocatalytic Activity of Novel p-Ag_3_PO_4_/n-BiFeO_3_ Heterojunction Composites for Dye Degradation. Nanoscale Res. Lett..

[28] Niu F., Chen D., Qin L., Zhang N., Wang J., Chen Z., Huang Y. (2015). Facile Synthesis of Highly Efficient p-n Heterojunction CuO/BiFeO_3_ Composite Photocatalysts with Enhanced Visible-Light Photocatalytic Activity. Chemcatchem.

[29] Soltani T., Tayyebi A., Lee B.-K. (2018). BiFeO_3_/BiVO_4_ p−n heterojunction for efficient and stable photocatalytic and photoelectrochemical water splitting under visible-light irradiation. Catal. Today.

[30] Hu X., Wang W., Xie G., Wang H., Tan X., Jin Q., Zhou D., Zhao Y. (2019). Ternary assembly of g-C_3_N_4_/graphene oxide sheets /BiFeO_3_ heterojunction with enhanced photoreduction of Cr(VI) under visible-light irradiation. Chemosphere.

[31] Theerthagiri J., Chandrasekaran S., Salla S., Elakkiya V., Senthil R.A., Nithyadharseni P., Maiyalagan T., Micheal K., Ayeshamariam A., Arasu M.V. (2018). Recent developments of metal oxide based heterostructures for photocatalytic applications towards environmental remediation. J. Solid State Chem..

[32] De Mendonça V.R., Dalmaschio C.J., Leite E.R., Niederberger M., Ribeiro C. (2015). Heterostructure formation from hydrothermal annealing of preformed nanocrystals. J. Mater. Chem. A.

[33] Wang F., Yang H., Zhang Y. (2018). Enhanced photocatalytic performance of CuBi_2_O_4_ particles decorated with Ag nanowires. Mater. Sci. Semicond. Process..

[34] Zhang Y.C., Yang H., Wang W.P., Zhang H.M., Li R.S., Wang X.X., Yu R.C. (2016). A promising supercapacitor electrode material of CuBi_2_O_4_ hierarchical microspheres synthesized via a coprecipitation route. J. Alloys Compd..

[35] Elaziouti A., Laouedj N., Bekka A. (2016). Synergetic effects of Sr-doped CuBi2O4 catalyst with enhanced photoactivity under UVA- light irradiation. Environ. Sci. Pollut. Res. Int..

[36] Duan Q., Kong F., Han X., Jiang Y., Liu T., Chang Y., Zhou L., Qin G., Zhang X. (2019). Synthesis and characterization of morphology-controllable BiFeO_3_ particles with efficient photocatalytic activity. Mater. Res. Bull..

[37] Li Y., Wang X.-T., Zhang X.-Q., Li X., Wang J., Wang C.-W. (2020). New hydrothermal synthesis strategy of nano-sized BiFeO_3_ for high-efficient photocatalytic applications. Phys. E Low Dimens. Syst. Nanostruct..

[38] Yuvaraj S., Karthikeyan K., Kalpana D., Lee Y.S., Selvan R.K. (2016). Surfactant-free hydrothermal synthesis of hierarchically structured spherical CuBi_2_O_4_ as negative electrodes for Li-ion hybrid capacitors. J. Colloid Interface Sci..

[39] Zhang F., Saxena S. (2006). Raman studies of Bi_2_CuO_4_ at high pressures. Appl. Phys. Lett..

[40] Popović Z., Kliche G., Cardona M., Liu R. (1990). Vibrational properties of Bi_2_CuO_4_. Phys. Rev. B Condens. Matter.

[41] Najafian H., Manteghi F., Beshkar F., Salavati-Niasari M. (2019). Fabrication of nanocomposite photocatalyst CuBi_2_O_4_/Bi_3_ClO_4_ for removal of acid brown 14 as water pollutant under visible light irradiation. J. Hazard. Mater..

[42] Gao H., Wang F., Wang S., Wang X., Yi Z., Yang H. (2019). Photocatalytic activity tuning in a novel Ag_2_S/CQDs/CuBi_2_O_4_ composite: Synthesis and photocatalytic mechanism. Mater. Res. Bull..

[43] Shi W., Guo F., Yuan S. (2017). In situ synthesis of z-scheme Ag_3_PO_4_/CuBi_2_O_4_ photocatalysts and enhanced photocatalytic performance for the degradation of tetracycline under visible light irradiation. Appl. Catal. B Environ..

[44] Yang J., Du C., Wen Y., Zhang Z., Cho K., Chen R., Shan B. (2018). Enhanced photoelectrochemical hydrogen evolution at p-type CuBi2O4 photocathode through hypoxic calcination. Int. J. Hydrog. Energy.

[45] Chen Y., Zhang Y., Luo L., Shi Y., Wang S., Li L., Long Y., Jiang F. (2017). A novel templated synthesis of C/N-doped β-Bi_2_O_3_ nanosheets for synergistic rapid removal of 17α-ethynylestradiol by adsorption and photocatalytic degradation. Ceram. Int..

[46] Yang S., Chen C., Liu L., Zhu L., Xu X. (2017). Facile fabrication of micro-floriated AgBr/Bi_2_O_3_ as highly efficient visible-light photocatalyst. Mater. Res. Bull..

[47] Claros M., Setka M., Jimenez Y.P., Vallejos S. (2020). AACVD Synthesis and Characterization of Iron and Copper Oxides Modified ZnO Structured Films. Nanomaterials.

[48] Zalecki R., Woch W.M., Kowalik M., Kolodziejczyk A., Gritzner G. (2010). Bismuth Valence in a Tl_0.7_Bi_0.3_Sr_1.6_Ba_0.4_CaCu_2_O_y_ Superconductor from X-Ray Photoemission Spectroscopy. Acta Phys. Pol. A.

[49] Huo Y., Jin Y., Zhang Y. (2010). Citric acid assisted solvothermal synthesis of BiFeO3 microspheres with high visible-light photocatalytic activity. J. Mol. Catal. A Chem..

[50] Luo W., Zhu L., Wang N., Tang H., Cao M., She Y. (2010). Efficient Removal of Organic Pollutants with Magnetic Nanoscaled BiFeO_3_ as a Reusable Heterogeneous Fenton-Like Catalyst. Environ. Sci. Technol..

[51] Di L., Yang H., Xian T., Chen X. (2018). Enhanced photocatalytic degradation activity of BiFeO_3_ microspheres by decoration with g-C_3_N_4_ nanoparticles. Mater. Res..

[52] Fatima S., Ali S.I., Younas D., Islam A., Akinwande D., Rizwan S. (2019). Graphene nanohybrids for enhanced catalytic activity and large surface area. MRS Commun..

[53] Jiang Z., Geng Y., Gu D. (2008). Write-once medium with BiO_x_ thin films for blue laser recording. Chin. Opt. Lett..

[54] Zuo W., Zhu W., Zhao D., Sun Y., Li Y., Liu J., Lou X.W.D. (2016). Bismuth oxide: A versatile high-capacity electrode material for rechargeable aqueous metal-ion batteries. Energy Environ. Sci..

