# Supplementary material for: One Step Synthesis of Tetragonal-CuBi2O4/Amorphous-BiFeO3 Heterojunction with Improved Charge Separation and Enhanced Photocatalytic Properties
Source: Nanomaterials (Basel). 2020 Aug 1;10(8):1514. doi: 10.3390/nano10081514 (PMC7466469; doi:10.3390/nano10081514)
Supplement: Supplementary file 1 [file nanomaterials-10-01514-s001.pdf]

Supplementary Materials

One step synthesis of tetragonal-$CuBi_2O_4$/amorphous-$BiFeO_3$ heterojunction with improved charge separation and enhanced photocatalytic properties

Fang Cai[1, 2, †], Ting Zhang[1, 3, †], Qiong Liu[4], Pengran Guo[1, 2], Yongqian Lei[1, 2], Yi Wang[3] and Fuxian Wang[1, 4*]

1 Guangdong Provincial Key Laboratory of Emergency Test for Dangerous Chemicals, Guangdong Institute of Analysis, Guangdong Academy of Sciences, Guangzhou 510070, China;
2 Guangdong Engineering Technology Research Center of On-line Monitoring of Water Environmental Pollution, Guangdong Institute of Analysis, Guangdong Academy of Sciences, Guangzhou 510070, China;
3 College of Petrochemical Technology, Lanzhou University of Technology, Lanzhou 730050, China;
4 State Key Laboratory of Pulp and Paper Engineering, South China University of Technology, Guangzhou 510641, China;
* Correspondence: wangfuxian@fenxi.com.cn (F.W.)
† These authors contributed equally to this work

Table S1 details for the preparation of solvothermal solutions with various ion ratios.

T-CBO/A-BFO ratio	Stock solution for each ion	n (mol)	V(ml)	C(mol/l)
1:4	BiN_3O_9 in acetic acid	0.00144	3.6	0.4
	$Cu(NO_3)_2{\cdot}3H_2O$ in ethanol	0.00024	6	0.04
	$Fe(NO_3)_3{\cdot}9H_2O$ in ethanol	0.00096	24	0.04
1:2	BiN_3O_9 in acetic acid	0.0016	4	0.4
	$Cu(NO_3)_2{\cdot}3H_2O$ in ethanol	0.0004	10	0.04
	$Fe(NO_3)_3{\cdot}9H_2O$ in ethanol	0.0008	20	0.04
1:1	BiN_3O_9 in acetic acid	0.0018	4.5	0.4
	$Cu(NO_3)_2{\cdot}3H_2O$ in ethanol	0.0006	15	0.04
	$Fe(NO_3)_3{\cdot}9H_2O$ in ethanol	0.0006	15	0.04
2:1	BiN_3O_9 in acetic acid	0.002	5	0.4
	$Cu(NO_3)_2{\cdot}3H_2O$ in ethanol	0.0008	20	0.04
	$Fe(NO_3)_3{\cdot}9H_2O$ in ethanol	0.0004	10	0.04
3:1	BiN_3O_9 in acetic acid	0.0021	5.25	0.4
	$Cu(NO_3)_2{\cdot}3H_2O$ in ethanol	0.0009	22.5	0.04
	$Fe(NO_3)_3{\cdot}9H_2O$ in ethanol	0.0003	7.5	0.04
4:1	BiN_3O_9 in acetic acid	0.00216	5.4	0.4
	$Cu(NO_3)_2{\cdot}3H_2O$ in ethanol	0.00096	24	0.04
	$Fe(NO_3)_3{\cdot}9H_2O$ in ethanol	0.00024	6	0.04

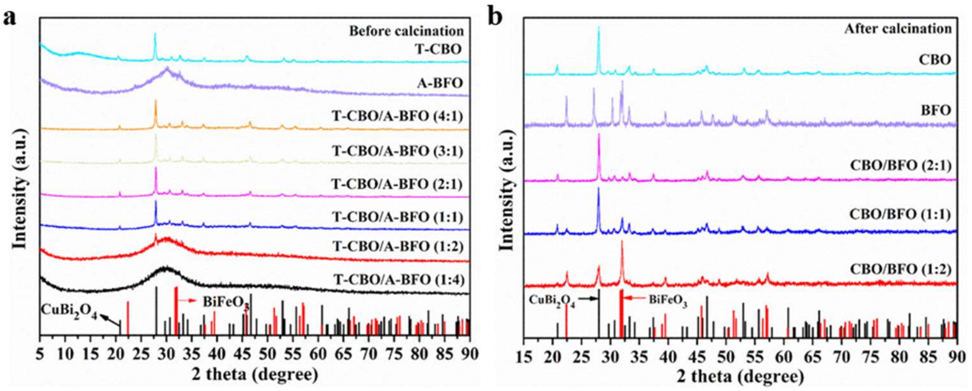

Figure S1 XRD patterns of the T-CBO, A-BFO and T-CBO/A-BFO composites (**a**) before annealing; (**b**) after annealing at 450 ℃ for 2 h.

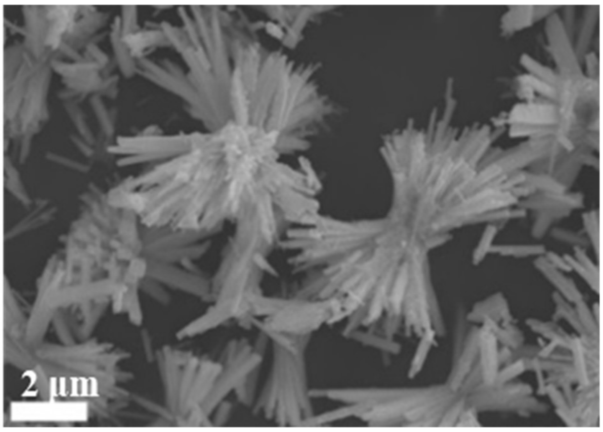

Figure S2. SEM images of the T-CBO.

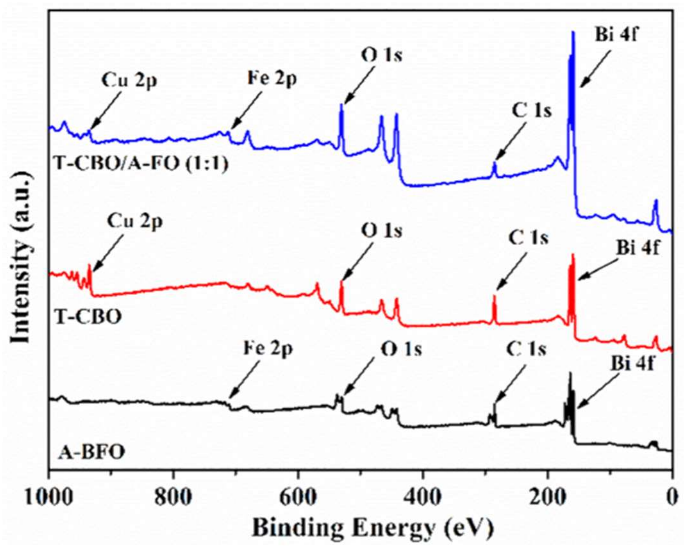

Figure S3. XPS survey spectra of Cu 2p, Fe 2p, O 1s, Bi 4f of the T-CBO, A-BFO and T-CBO/A-BFO (1:1).

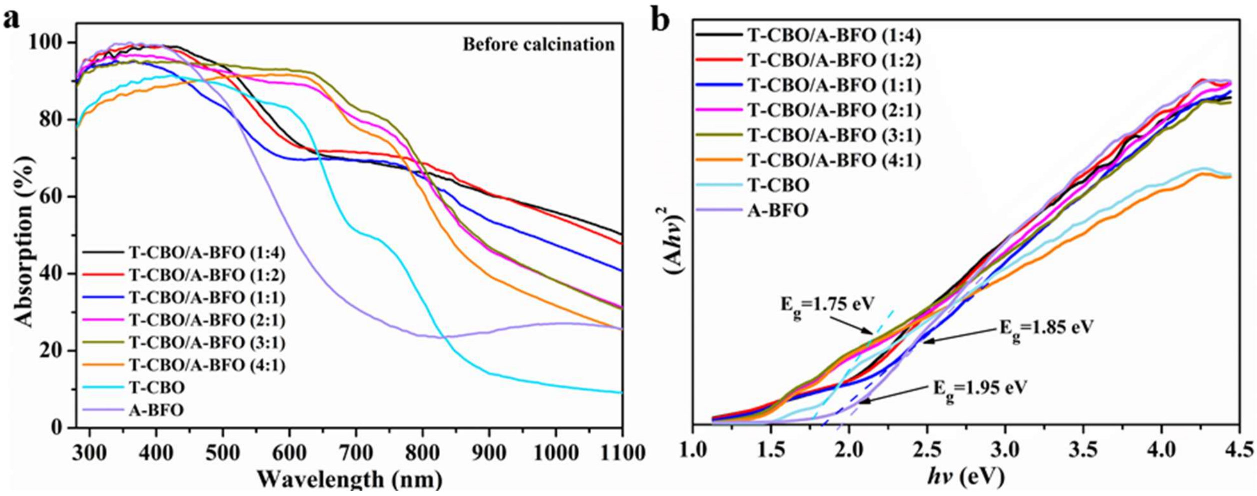

Figure S4. (**a**) UV-vis diffuse reflectance spectra; (**b**) corresponding Tauc plots of the T-CBO, A-BFO and T-CBO/A-BFO composites.

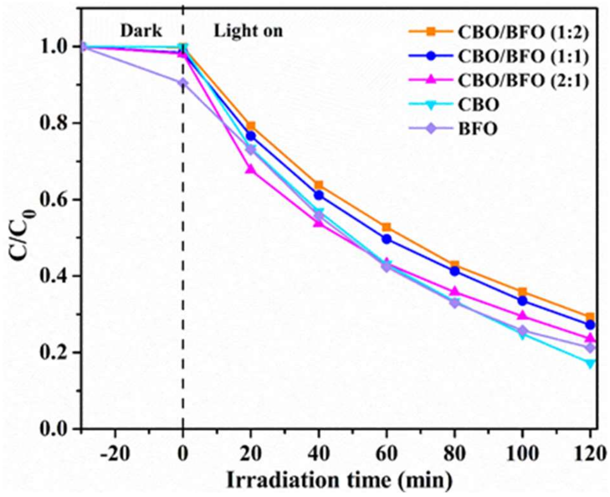

Figure S5. Photodegradation of MB by post annealed CBO, BFO and CBO/BFO composites.

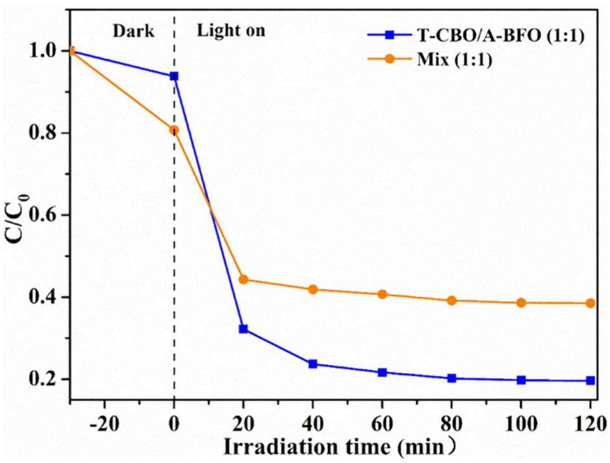

Figure S6. Photodegradation of MO by T-CBO/A-BFO (1:1) and the physically mixed T-CBO and A-BFO at a ratio of 1:1.
